# Humoral immunity induced by mRNA COVID-19 vaccines in Nursing Home Residents previously infected with SARS-CoV-2

**DOI:** 10.1007/s40520-022-02239-0

**Published:** 2022-09-21

**Authors:** Giorgio Fedele, Annapina Palmieri, Cecilia Damiano, Anna Di Lonardo, Pasqualina Leone, Ilaria Schiavoni, Caterina Trevisan, Angela Marie Abbatecola, Carmine Cafariello, Alba Malara, Pasquale Minchella, Giuseppina Panduri, Raffaele Antonelli Incalzi, Anna Teresa Palamara, Paola Stefanelli, Graziano Onder, The GeroCovid Vax Study Group

**Affiliations:** 1 VPD, Reference Labs Unit, Department of Infectious Diseases, Istituto Superiore di Sanità, Rome, Italy; 2 Department of Cardiovascular, Endocrine-Metabolic Diseases and Aging, Istituto Superiore di Sanità, Rome, Italy; 3 Department of Medical Sciences, University of Ferrara, Ferrara, Italy; 4 Alzheimer’s Disease Day Clinic Department, Azienda Sanitaria Locale, Frosinone, Italy; 5 Geriatrics Outpatient Clinic and Territorial Residences, Italian Hospital Group, Rome, Italy; 6 ANASTE Humanitas Foundation, Rome, Italy; 7 Department of Microbiology and Virology, Pugliese Ciaccio Hospital, Catanzaro, Italy; 8 Geriatrics Unit, Department of Medicine, Campus Bio-Medico University and Teaching Hospital, Rome, Italy

**Keywords:** SARS-CoV-2, COVID-19 vaccines, Nursing homes, Frailty

## Abstract

**Background:**

Nursing home (NH) residents suffered the greatest impact of the COVID-19 pandemic. Limited data are available on vaccine-induced immunity and on the protection ensured by a prior infection in this population.

**Aims:**

The present study aims to monitor antibody levels and their persistence over a 6-month period in NH residents according to the history of prior SARS-CoV-2 infection.

**Methods:**

We measured anti-trimeric Spike IgG antibody levels in a sample of 395 residents from 25 NHs in 6 Italian Regions at study enrolment (prior to the first dose of vaccine, T0) and then after 2 (T1) and 6 months (T2) following the first vaccine dose. All participants received mRNA vaccines (BNT162b2 or mRNA-1273). Analyses were performed using log-transformed values of antibody concentrations and geometric means (GM) were calculated.

**Results:**

Superior humoral immunity was induced in NH residents with previous SARS-CoV-2 infection. (T0: GM 186.6 vs. 6.1 BAU/ml, *p* < 0.001; T1: GM 5264.1 vs. 944.4 BAU/ml, *p* < 0.001; T2: GM 1473.6 vs. 128.7 BAU/ml, *p* < 0.001). Residents with prior SARS-CoV-2 infection receiving two vaccine doses presented significantly higher antibody concentration at T1 and T2. A longer interval between previous infection and vaccination was associated with a better antibody response over time.

**Discussion:**

In a frail sample of NH residents, prior SARS-CoV-2 infection was associated with a higher humoral response to vaccination. Number of vaccine doses and the interval between infection and vaccination are relevant parameters in determining humoral immunity.

**Conclusions:**

These findings provide important information to plan future immunization policies and disease prevention strategies in a highly vulnerable population.

## Introduction

Coronavirus Disease (COVID-19) pandemic caused by severe acute respiratory syndrome coronavirus 2 (SARS-CoV-2) has determined a substantial increment in morbidity and mortality worldwide [1]. Nursing home (NH) residents, that often present with a high burden of comorbidities and clinical complexities, suffered the greatest impact of the pandemic in many countries worldwide, independently of health care system organization [2–5]. Epidemiological data indicate that during the first pandemic wave, up to 50% of deaths from COVID-19 may have occurred within NH facilities [6]. For this reason, in many Western countries, priority has been given to this population to receive anti-SARS-CoV-2 vaccination. Starting from the end of December 2020, the Italian government has implemented vaccination programmes in NH to reduce the risk of COVID-19-related morbidity and mortality in this high-risk population [7]. Priority was given to the use of mRNA vaccines (mRNA-1273 or BNT162b2) in this population. Residents with no prior history of SARS-CoV-2 infection received two doses of mRNA vaccine. Vaccination was also recommended for residents with a history of prior SARS-CoV-2 infection. The Italian Ministry of Health recommended a single dose for those with a history of SARS-CoV-2 infection between 3 and 6 months beforehand and two doses for those with a history of infection more than 6 months beforehand [7].

Recent data indicate that immune response to vaccination tends to reduce over time and that this reduction is associated with breakthrough SARS-CoV-2 infections [8–10]. In addition, in healthy individuals, a prior SARS-CoV-2 infection was associated with a higher antibody level, suggesting that prior infection history may increase protection from vaccination and that different strategies and timing could be employed in the vaccination strategy, as well as in the administration of booster doses, in relation to the history of previous infection [11]. However, the available evidence cannot be generalized to NH residents. Limited data are available on the duration of the protection of vaccination and on the immunisation, coverage ensured by a prior infection in this population. In addition, immune response to vaccination might be reduced in frail NH residents with clinical complexity and it might be hypothesized that the potential benefits of hybrid immunity may also be decreased in this population compared to healthy individuals.

For these reasons, the present study aims to monitor antibody levels and their persistence over a 6-months period in NH residents according to the history of prior SARS-CoV-2 infection. This study is performed in the context of the GeroCovid Vax study [12], a multicentre Italian project monitoring effectiveness of SARS-CoV-2 vaccination in NH nationwide.

## Methods

### GeroCovid Vax study

GeroCOVID VAX study is promoted by the Italian Society of Gerontology and Geriatrics (SIGG) (Florence, Italy) and the Istituto Superiore di Sanità (ISS, Rome, Italy) and sponsored by the Italian Medicines Agency (AIFA). The study began in February 2021 and aimed at investigating the effects of anti-SARS-CoV-2 vaccine use in older NH residents in Italy [12]. Residents were consecutively enrolled within the vaccination calendar scheduled in each participating NH. The delay with respect to the formal start of the vaccination campaign had no impact on the representativeness of the study sample as the vaccination become fully operational in the first quarter of 2021. Main GeroCovid Vax study objectives include: (i) evaluating he efficacy and safety of the SARS-CoV-2 vaccine in a representative sample of older NH residents, and (ii) evaluate humoral and cellular immune response in a large subpopulation of study participants. The inclusion criteria were age ≥ 60 years, life expectancy ≥ 3 months, expected NH stay ≥ 3 months, receiving at least one dose of any type of anti-SARS-CoV-2 vaccine. Demographic and clinical data were collected by trained researchers at study enrolment (prior to the first dose of vaccine). These included age, sex, chronic diseases, and history of confirmed SARS-CoV-2 infection (as assessed by reverse transcriptase–polymerase chain reaction testing). Humoral immune response was evaluated in a sample of 395 residents at study enrolment (prior to the first dose of vaccine, T0) and then 2 (T1) and 6 months (T2) following the first vaccine dose.

### Serum preparation and storage

All blood samples were properly prepared and stored following a standardized procedure. Blood samples were collected in Serum Separator Tubes (BD Diagnostic Systems, Franklin Lakes, NJ, USA) and centrifuged at room temperature at 1600 rpm for 10 min. Aliquots were transferred to 2 ml polypropylene screw cap cryotubes (Nunc™, Thermofisher Scientific, Waltham, MA USA) and immediately frozen at -20 °C. Frozen sera were then shipped to the ISS as a national reference laboratory for COVID-19, in dry ice following biosafety shipment condition. Upon arrival serum samples were immediately stored at − 80 °C.

### SARS-CoV-2 IgG immunoassays

The Liaison® SARS-CoV-2 TrimericS IgG chemiluminescent assay (DiaSorin, Italy), using the trimeric S antigen stabilized in its native form and designed for high throughput in healthcare settings was used. The LIAISON® XL fully automated chemiluminescence analyzer automatically calculates SARS-CoV-2 trimeric S IgG antibody concentrations, expressed as binding antibody units (BAU/ml). The assay range is up to 2080 BAU/ml. According to the manufacturer’s instructions, values ≥ 33.8 BAU/ml were interpreted as positive. Samples that were above the upper limit of the assay were automatically diluted 1:20 and re-analysed.

### Statistical analysis

Baseline characteristics according to prior SARS-CoV-2 infection were compared using analysis of variance (ANOVA) for normally distributed variables, nonparametric Kruskal–Wallis *H* tests for skewed variables and chi-square analyses for dichotomous variables. Given the non-normal distribution of SARS-CoV-2 trimeric S IgG antibody concentrations, analyses were performed using log-transformed values and geometric means were calculated (GM). Analysis of covariance (ANCOVA) was used to compare means of SARS-CoV-2 trimeric S IgG antibody concentrations according to prior SARS-CoV-2 infection. Among residents with prior SARS-CoV-2 infection antibody concentration according to number of doses received (1 vs. 2 doses) and time from SARS-CoV-2 infection (≤ 4 months vs. ≥ 5 months) was calculated. To avoid the confounding effect related to the number of vaccine doses received, the effect of time from SARS-CoV-2 infection on antibody concentration was analysed only in residents receiving a single vaccine dose. Variables considered for adjustment in the ANCOVA models were age, sex and those associated with prior SARS-CoV-2 infection at *p* ≤ 0.10 at the univariate analysis. Statistical analysis was carried out with STATA Software Version 16.1 (Stata Cooperation, College Station, TX, USA).

### Ethical approval

The study was approved by the Italian National Ethical Committee with permission number 264/2021 (January 26, 2021).

## Results

### Study sample

The study sample consisted of 395 residents from 25 NHs in 6 Italian Regions. Table 1 shows resident’s characteristics according to prior SARS-CoV-2 infection. Mean age of the study sample was 82.4 ± 9.5 years and 68% of residents were women. Most residents received BNT162b2 vaccine (87%). Dementia was the most common condition observed (54% of residents), followed by hypertension (50%), ischemic heart diseases (29%), diabetes (16%) and chronic obstructive pulmonary disease (16%). Overall, 139 residents (35.2%) had SARS-CoV-2 infection prior to study enrolment. As compared with residents with prior SARS-CoV-2 infection, those without infection were older and had a significantly higher prevalence of chronic renal failure. Among the 139 residents with prior SARS-CoV-2 infection, 118 (85%) received a single vaccine dose and 21 (15%) two doses. Among the 118 residents receiving a single dose of vaccine, 80 (67.8%) had prior infection ≤ 4 months before vaccination, 33 (28.0%) ≥ 5 months before vaccination and in 5 (4.2%) residents the information on SARS-CoV-2 infection date was not available.

**Table 1:** Demographic and clinical characteristics of the study sample

		Whole sample (*n* = 395)	Prior SARS-CoV-2 infection (*n* = 139)	No prior SARS-CoV-2 infection (*n* = 256)	*p* value
Age	< 80 years	131 (33%)	63 (45%)	68 (27%)	< 0.001
	≥ 80 years	264 (67%)	76 (55%)	188 (73%)	
Sex	Female	270 (68%)	91 (65%)	179 (70%)	0.4
	Male	125 (32%)	48 (35%)	77 (30%)	
Type of vaccine	BNT162b2	344 (87%)	117 (84%)	227 (89%)	0.6
	mRNA-1273	51 (13%)	22 (16%)	29 (11%)	
Chronic diseases	Dementia	213 (54%)	88 (63%)	125 (49%)	0.58
	Arterial Hypertension	201 (51%)	69 (50%)	132 (51%)	0.7
	Ischemic Heart Disease	114 (29%)	35 (25%)	79 (31%)	0.2
	Diabetes	65 (16%)	21 (15%)	44 (17%)	0.1
	COPD	63 (16%)	27 (19%)	36 (14%)	0.7
	Atrial fibrillation	30 (7.5%)	8 (6%)	22 (9%)	0.6
	Stroke	43 (11%)	19 (14%)	24 (9%)	0.6
	Chronic Renal Failure	46 (12%)	9 (6%)	37 (14%)	0.02
	Cardiac failure	29 (7%)	8 (6%)	21 (8%)	0.13
	Obesity	27 (7%)	11 (8%)	16 (6%)	0.9
	Cancer	23 (6%)	10 (7%)	13 (5%)	0.9
	Chronic Liver Disease	18 (5%)	5 (4%)	13 (5%)	0.2
	Immune System Disorder	11 (3%)	6 (4%)	5 (2%)	0.4

*COPD* Chronic obstructive pulmonary disease

### SARS-CoV-2 trimeric S IgG antibody concentration and prior-SARS-CoV-2 infection

**Fig. 1:**
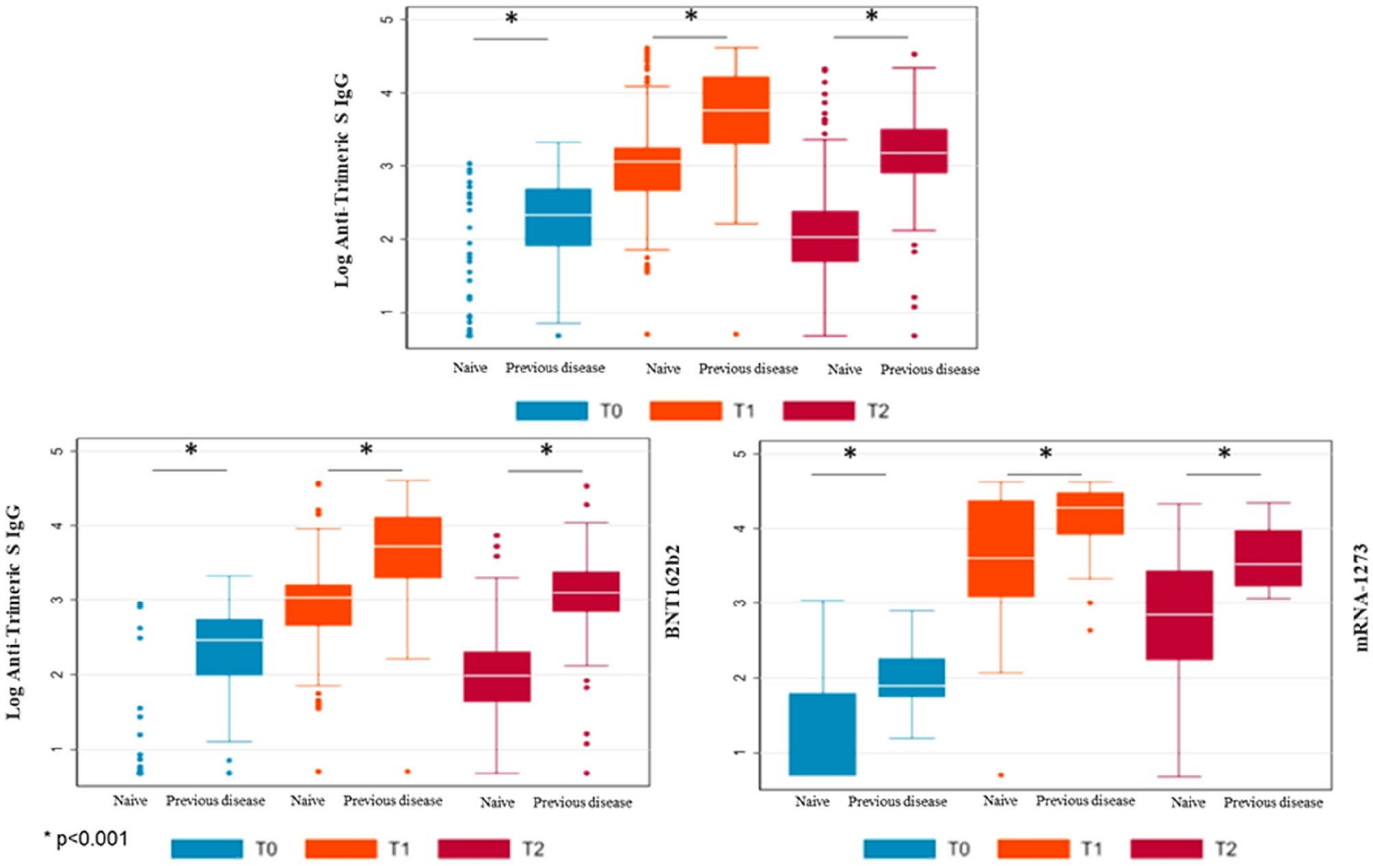
SARS-CoV-2 trimeric S IgG antibody concentration (log-transformed values) before vaccination (T0), 2 months (T1) and 6 months (T2) after first dose of vaccine according to prior SARS-CoV-2 infection. Data are presented for the whole sample (upper panel, *n* = 395), for BNT162b2 (*n* = 344, lower left panel), and mRNA-1273 vaccines (*n* = 51 lower right panel)

Figure 1 shows SARS-CoV-2 trimeric S IgG antibody concentration before vaccination (T0), 2 months (T1) and 6 months (T2) after the first dose according to prior SARS-CoV-2 infection. Significantly higher antibody levels were present at T0 in residents with prior SARS-CoV-2 infection. Antibody concentration reached the highest level 2 months after vaccination (T1) and then declined at 6 months (T2).

Overall, 392 (99%) residents at 2 months (T1) and 343 (87%) at 6 months after vaccination (T2) had an antibody concentration above the 33.8 BAU/ml threshold. The response to vaccination was significantly higher in residents with prior SARS-CoV-2 infection who continued to have significantly higher antibody levels at 2- (T1) and 6-month (T2) follow-ups. When data were stratified according to the type of vaccine, we found that the effect of prior SARS-CoV-2 infection on antibody concentration was consistent for both mRNA-1273 and BNT162b2 vaccines. We did not observe any significant impact of any of the conditions suffered by residents reported on the antibody response to vaccination.

Table 2 shows adjusted GM (Standard Errors) of SARS-CoV-2 trimeric S IgG antibody concentration among participating residents, according to prior SARS-CoV-2 infection. Participants with a prior infection presented a significant higher concentration of antibodies than those without a history of infection before vaccination (T0; GM 186.6 vs. 6.1 BAU/ml, *p* < 0.001) and at the 2- (T1; GM 5264.1 vs. 944.4 BAU/ml, *p* < 0.001) and 6-month (T2; GM 1473.6 vs. 128.7 BAU/ml, *p* < 0.001) follow-ups. This association was consistent in both users of mRNA-1273 or BNT162b2. Independently of prior SARS-CoV-2 infection, residents receiving mRNA-1273 had significantly higher antibody concentration than those receiving BNT162b2.

**Table 2:** Geometric means (Standard Errors) of Anti-S IgG (BAU/mL), according to prior SARS-CoV-2 infection, at baseline assessment (before vaccination—T0), 2 months (T1) and 6 months (T2) after first dose of vaccine

	Anti-S IgG serum concentration, Geometric Means (SE), BAU/mL								
	Before vaccination (T0)			2 months after first dose (T1)			6 months after first dose (T2)		
	Geometric mean	SE	*p* value	Geometric mean	SE	*p* value	Geometric mean	SE	*p* value
Whole sample									
Prior SARS-CoV-2 infection (*n* = 139)	186.6	19.6	< 0.001	5264.1	724.6	< 0.001	1473.6	203.1	< 0.001
No prior SARS-CoV-2 infection (*n* = 256)	6.1	0.6		944.4	111.3		128.7	15.2	
*BNT162b2*									
Prior SARS-CoV-2 infection (*n* = 117)	219.9	21.4	< 0.001	4838.2	651.7	< 0.001	1225.2	159.5	< 0.001
No prior SARS-CoV-2 infection (*n* = 227)	5.3	0.4		827.6	93.7		107.1	11.7	
*mRNA-1273*									
Prior SARS-CoV-2 infection (*n* = 29)	88.6	37.4	0.001	8949.7	4828.6	0.1	5085.3	2547.7	< 0.001
No prior SARS-CoV-2 infection (*n* = 22)	20.6	7.9		3636.3	1796.7		893.9	410.2	

Analyses are adjusted by age, sex and chronic renal failure

*SE* standard error

### SARS-CoV-2 trimeric S IgG antibody concentration in residents with prior-SARS-CoV-2 infection

Table 3 shows geometric means of SARS-CoV-2 trimeric S IgG antibody concentration in residents with prior SARS-CoV-2 infection according to number of doses received and time of SARS-CoV-2 infection diagnosis. Residents with prior SARS-CoV-2 infection receiving two vaccine doses presented significantly higher antibody concentration in the 2- and 6-month follow-ups (T1 and T2). Independently of the number of doses received, residents with prior SARS-CoV-2 infection had higher antibody concentration at 2- and 6-month follow-ups than those with no SARS-CoV-2 infection history. To avoid the confounding effect related to the number of vaccine doses received, the effect of time from SARS-CoV-2 infection on antibody concentration was analysed only in residents receiving a single vaccine dose. As shown in Table 3, residents experiencing SARS-CoV-2 infection ≥ 5 months before vaccination had a lower level of antibody concentration before vaccination (T0), but this association was reversed after vaccination. At the 2- and 6-month follow-ups (T1 and T2), this group presented a significantly higher antibody concentration as compared with residents experiencing SARS-CoV-2 infection ≤ 4 months before vaccination. The multiplicative factor of a previous infection on the antibody response induced by vaccination was 11.4 at the T2 follow-up. More in detail, SARS-CoV-2 trimeric S IgG antibody concentration after a single vaccine dose, was 16.1-fold higher in residents who had the previous infection ≥ 5 months before vaccination and 7.3-fold higher in residents who had SARS-CoV-2 infection ≤ 4 months before vaccination.

**Table 3:** Geometric means (Standard Errors) of Anti-S IgG (BAU/mL) in residents with Prior SARS-CoV-2 infection at baseline assessment (before vaccination—T0), 2 months (T1) and 6 months (T2) after the first dose of vaccine according to number of doses received and months from SARS-CoV-2 infection

	Anti-S IgG serum concentration, Geometric Means (SE), BAU/mL									
	Before vaccination (T0)				2 months after first dose (T1)			6 months after first dose (T2)		
	Geometric mean		SE	*p* value	Geometric mean	SE	*p* value	Geometric mean	SE	*p* value
Prior SARS-CoV-2 infection										
Number of doses										
1 dose (*n* = 118)	207.5		31.5	0.004	4400.7	696.7	0.02	1182.9	175.2	0.006
2 doses (*n* = 21)	80.8		25.7		9639.6	3204.2		2847.4	885.4	
Prior SARS-CoV-2 infection and 1 vaccine dose										
Months from diagnosis										
≤ 4 months (*n* = 80)		233.6	41.2	0.05	4109.8	738.5	0.02	938.9	160.2	0.001
≥ 5 months (*n* = 33)		147.7	30.7		7108.7	1496.0		2074.2	414.5	

Analyses are adjusted by age, sex and chronic renal failure

*SE *Standard Error

## Discussion

Frail NH residents suffered the most severe consequences from SARS-CoV-2 epidemic. For this reason, particular attention to the effect of prevention strategies on this population is a priority to limit the impact of the epidemic worldwide. The main finding of the present study is that significantly superior humoral immunity is induced by mRNA vaccines in NH residents with a previous SARS-CoV-2 infection compared to residents without prior infection.

Overall, the administration of SARS-CoV-2 mRNA vaccines resulted immunogenic in NH residents. Indeed, two months after the completion of the primary vaccine schedule, almost 100% of the residents showed a positive anti-trimeric Spike IgG titer. Six months after the first immunization still more than 85% of residents had a titer above the positivity cut-off of the serological assay used, an observation that extends to older adults living in NH findings results obtained in other settings and age-groups [8–11]. The number of vaccine doses influenced the antibody response. A longer interval between SARS-CoV-2 infection and vaccination was associated with a better humoral immune response.

It has been shown that in subjects previously infected by SARS-CoV-2, vaccination increased all humoral response components [13]. The potentiating effect of a previous infection is not surprising and could be attributed to the so-called hybrid immunity [14]. Immunological memory generated after exposure to SARS-CoV-2 is promptly activated by subsequent vaccine immunization, leading to an enhanced immune response [14].

The hybrid immunity effect on the magnitude of vaccine-induced humoral response has been shown in several studies. Among vaccinated health care workers from the UK, previously infected individuals expressed higher levels of anti-SARS-CoV-2 IgG than those with no previous SARS-CoV-2 infection after a single dose of BNT162b2 [15]. Abu Jabal et al. reported that individuals with previous SARS-CoV-2 infection had antibody titers one order of magnitude higher than those without a previous infection independently of ethnicity or sex [16]. An additional study by Ebinger et al. found that, among previously infected individuals, a single dose of BNT162b2 induced a similar antibody response as compared to the response measured in naïve subjects after two doses of vaccine [17]. Most important, recent studies in NH residents with or without a previous history of SARS-CoV-2 infection, have shown higher antibody response ensured by the hybrid immunity effect. Martinek and colleagues showed that 5–7 months after vaccination immune parameters were significantly higher in convalescent residents than in naive residents after vaccination [18]. Similar findings were described by Jeulin et al. who showed that NH residents with a history of SARS-CoV-2 infection have a clear advantage in the magnitude and duration anti-S IgG titers following the 2nd dose [19]. The finding of the beneficial hybrid immunity effect in NH residents is of relevance when considering the characteristics of the population under study. In fact, it has been postulated that frail older people experience defects and impairments of their immune response [20] which may result in the inability to mount effective vaccine responses [21, 22].

We found that a longer interval between previous SARS-CoV-2 infection and vaccination is associated with a higher antibody response at two and six months after the immunization. This observation is in line with a previous study [15] and consistent with works showing the enhanced immunogenicity of a two-dose regimen SARS-CoV-2 vaccination with an extended interval [23–25]. Considering that the first vaccine dose in previously infected residents acts as a boost for the immune memory pools generated after the infection, the longer interval from the infection may have been instrumental in building optimal B and T cell memory pools [26].

Despite the reduction of antibody titers between T1 and T2, most residents were still seropositive, in line with observations showing that vaccine-induced antibodies may last for several months after vaccination [9, 27]. The slower decline in anti-Trimeric S IgG levels over time in residents with a previous SARS-CoV-2 infection is consistent with results from previous studies and confirms the effect of the previous infection in enhancing immune response [28, 29].

The humoral response to vaccination was significantly higher in recipients of the mRNA-1273 vaccine, either in previously infected or naïve subjects. This observation confirms previous studies [30] and might be explained by the higher mRNA content in mRNA-1273 compared with BNT162b2.

This study has some limitations. The follow-up of the study is limited to 6 months and it is not possible to assume any effect of vaccination or hybrid immunity beyond this period. In addition, cell-mediated immunity, which is stimulated by vaccination and plays an important role in protecting against SARS-CoV-2 infection, was not measured. Finally, the effect of possible SARS-CoV-2 infections occurring during the follow-up was not taken into account. The major strength of the study is related to the fact that it focused on a population that is often neglected by clinical studies, but which has suffered the greatest impact in terms of mortality and morbidity from the epidemic.

Overall, the findings of the study provide relevant information potentially helping in driving future immunization policies and disease prevention strategies in the NH settings. Prior SARS-CoV-2 infection is associated with a higher humoral response to vaccination and the humoral response seems stronger in residents with a history of SARS-CoV-2 occurring ≥ 5 months before vaccination. These findings suggest that not only the number of doses but also the time of vaccination represent relevant parameters to be considered to maximize benefits of COVID-19 immunization.
